# Potential Effects of Climate Change on the Distribution of Cold-Tolerant Evergreen Broadleaved Woody Plants in the Korean Peninsula

**DOI:** 10.1371/journal.pone.0134043

**Published:** 2015-08-11

**Authors:** Kyung Ah Koo, Woo-Seok Kong, Nathan P. Nibbelink, Charles S. Hopkinson, Joon Ho Lee

**Affiliations:** 1 National Institute of Ecology, Seocheon-gun, Chungnam, Republic of Korea; 2 Kyung Hee University, Seoul, Republic of Korea; 3 University of Georgia, Athens, Georgia, United States of America; DOE Pacific Northwest National Laboratory, UNITED STATES

## Abstract

Climate change has caused shifts in species’ ranges and extinctions of high-latitude and altitude species. Most cold-tolerant evergreen broadleaved woody plants (shortened to cold-evergreens below) are rare species occurring in a few sites in the alpine and subalpine zones in the Korean Peninsula. The aim of this research is to 1) identify climate factors controlling the range of cold-evergreens in the Korean Peninsula; and 2) predict the climate change effects on the range of cold-evergreens. We used multimodel inference based on combinations of climate variables to develop distribution models of cold-evergreens at a physiognomic-level. Presence/absence data of 12 species at 204 sites and 6 climatic factors, selected from among 23 candidate variables, were used for modeling. Model uncertainty was estimated by mapping a total variance calculated by adding the weighted average of within-model variation to the between-model variation. The range of cold-evergreens and model performance were validated by true skill statistics, the receiver operating characteristic curve and the kappa statistic. Climate change effects on the cold-evergreens were predicted according to the RCP 4.5 and RCP 8.5 scenarios. Multimodel inference approach excellently projected the spatial distribution of cold-evergreens (AUC = 0.95, kappa = 0.62 and TSS = 0.77). Temperature was a dominant factor in model-average estimates, while precipitation was minor. The climatic suitability increased from the southwest, lowland areas, to the northeast, high mountains. The range of cold-evergreens declined under climate change. Mountain-tops in the south and most of the area in the north remained suitable in 2050 and 2070 under the RCP 4.5 projection and 2050 under the RCP 8.5 projection. Only high-elevations in the northeastern Peninsula remained suitable under the RCP 8.5 projection. A northward and upper-elevational range shift indicates change in species composition at the alpine and subalpine ecosystems in the Korean Peninsula.

## Introduction

Global climate change is a pivotal environmental issue for human well-being, economic growth, and nature conservation [1]. Much research has reported that climate change has caused changes in phenological events, species’ range shifts, and habitat loss of high-latitude and altitude species [2–9]. It was predicted that plants in Arctic and alpine regions would advance timing of spring phenological events under climate changes [10]. On the other hand, warmer temperature in fall and winter delayed timing of bud burst in the spring due to chilling deficiency [3]. Long-term ecological research in mountain ecosystems has showed the upward and poleward range expansion or migration of plant species under climate change [11–14]. Herbaceous species in Niwot Rigde, CO, USA showed the upward expansion or migration [14]. Studies implemented in European mountain ranges reported the upward range shift of plants from lower elevations showed an overall reduction in the European mountain flora [11–13]. Thermophilous species were relatively successful in colonizing high summits, but the cold-adapted species declined in the European mountain system [15–18]. Physiological research supported negative effects of warmer temperatures on annual growth and seedling and sapling establishment for alpine and subalpine species [19, 20]. Tropical plants also shifted their range upslope and higher latitude under climate change, which decreased species diversity in lower elevations [2, 5, 8, 9, 21].

Acclimation and adaptation potentials will determine the ecological response of plants to climate change and buffer the change effects on species distribution [22]. Plants acclimated their respiration to warming climate, regardless of the type of habitats such as alpine, tundra, lowland and tropical ecosystem [23, 24]. However, some studies showed that alpine species showed lower acclimation ability of photosynthesis and leaf respiration to warmer growth temperature than lowland plants [25, 26]. Adaptation potential to climate change relies on genetic diversity [22]. High genetic diversity enables plants to cope with climate warming through genetic differentiations among populations along a climatic gradient, as a consequence of environmental selection [22]. High genetic variations among populations along an altitudinal gradient were found in widespread alpine and subalpine plant species [27, 28]. Alpine and subalpine, rare, relict species, however, showed no or very low genetic variation within and among populations, which may indicate low adaptation potential to the warming [29]. Therefore, rare and relict alpine and subalpine species, which may have low acclimation potential, are especially vulnerable to climate warming.

The cold-tolerant evergreen broadleaved woody plants (shortened to cold-evergreens below) in the Korean Peninsula are distributed in the high mountains and North Korea. Most cold-evergreens are rare and relict dwarf shrubs occurring in a few sites in the alpine and subalpine zones [30]. It has been observed that alpine and subalpine dwarf shrub communities in Scotland, UK, have declined under climatic warming in interactions with anthropogenic environmental change [31–33]. The fifty-year survey in Scotland, UK, quantitatively showed that alpine and subalpine dwarf shrubs declined under climate changes [31]. We expect a decline in areal extent of suitable habitats for the cold-evergreens with climate change, as has been observed for other alpine and subalpine, rare, and relict dwarf shrubs [11, 16, 31]. We find that little research has investigated the most influential climatic factors controlling the distributions of cold-evergreen species in the Korean Peninsula despite the need for such information to support long-term management. The goals of this study are: 1) to determine the climate factors that best explain the distribution of cold-tolerant evergreen broadleaved woody plants in the Korean Peninsula; and 2) to predict the effects of potential climate change on species distribution. We hypothesize that: 1) warmer temperatures and drought stress will have negative effects on the distribution of cold-evergreens in the Korean Peninsula, and 2) the range of cold-evergreens will decline under the future climate change.

Species distribution modeling (SDM) is commonly applied to predict spatiotemporal variations in plant and animal distributions [34–36]. SDMs result in spatial projections (maps) indicating locations of the most suitable habitats for a target species and/or community [37]. Correlative SDMs have been widely used to predict species distributions due to the tractability of data requirements and modeling methods along with various free software programs [35–37]. This study employs multimodel inference (MMI) approach (Burnham and Anderson 2002). Model-averaged parameter estimates have been used for providing a relatively more stabilized inference than other approaches using single models for inference such as GLMs and CART [38].

## Methods

### Study area

**Fig 1:**
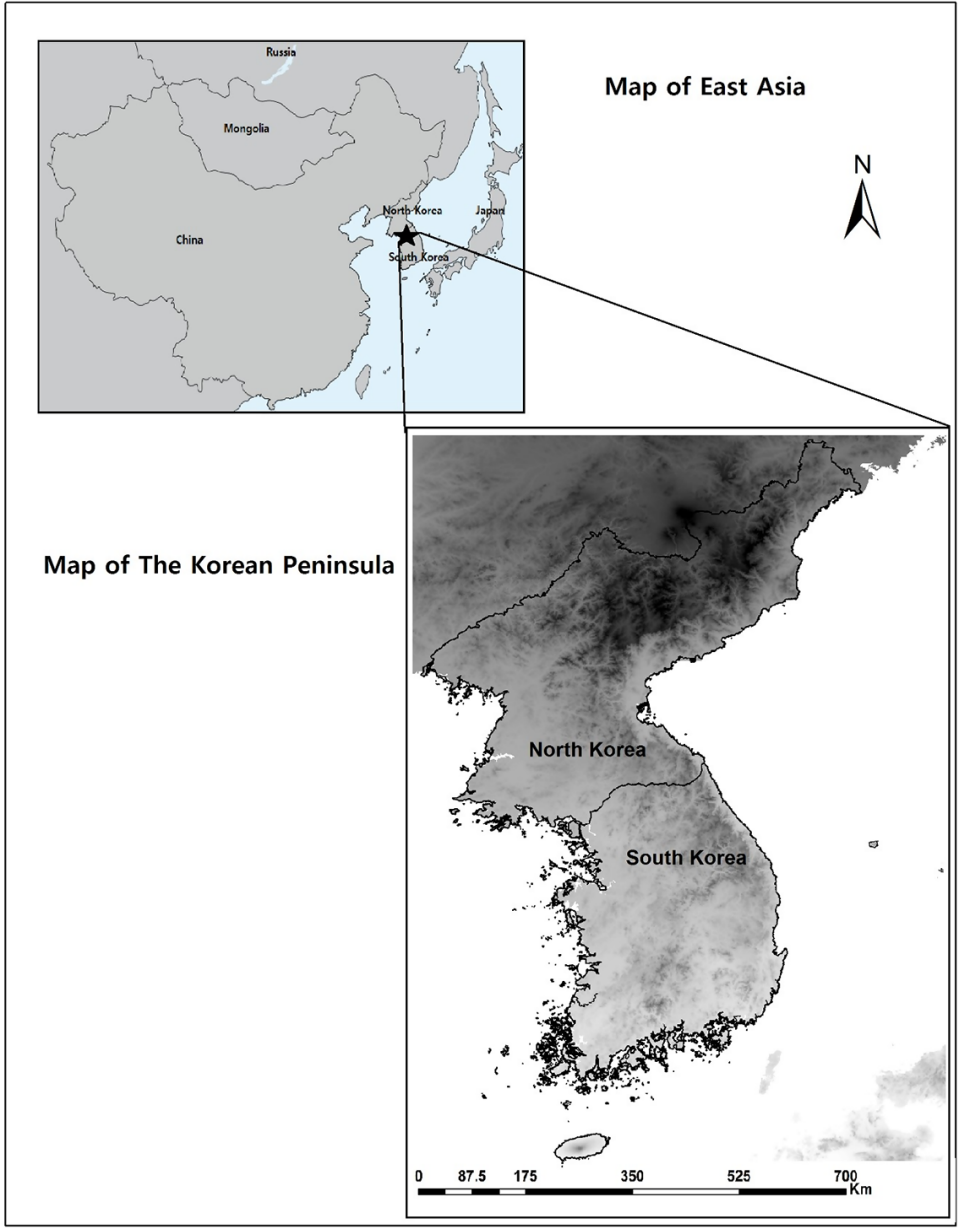
Map of the Korean Peninsula in the East Asia. The map of East Asia was obtained from www.thoughtyoumayask.com and then modified. The latitudinal range of Korean Peninsula is 33°N to 43°N and the longitudinal range 124°E to 132°E.

The study was conducted on the Korean Peninsula of the Republic of Korea (ROK) in the south and Democratic People's Republic of Korea (DPRK) in the north (Fig 1). The Peninsula has been well described in the literature [39], and the followings are summarized from Kong & Watts (1993). Briefly, the total area of the Peninsula is 220,847 km², which is approximately 70% mountainous and includes over 3,400 islands. The southern and western parts of the peninsula have well-developed plains, while the eastern and northern parts are mountainous regions. Roughly 300 kilometers in width, climate variations are more pronounced along the south-north axis, showing marked differences in climate and plant vegetation along this axis. The southern regions are relatively warm and wet, while the northern regions experience a colder and more continental climate. However, the entire peninsula is affected by similar general patterns, including the East Asian monsoon in midsummer and the frequent incidence of typhoons in autumn. Most rainfall takes place during summer, with nearly half during the monsoon alone. Winters are cold, with January temperatures typically below freezing, and winter precipitation is minimal, with little snow accumulation outside of mountainous areas. More than 3,500 plant species, including more than 500 endemics, have been identified in the peninsula [39]. The peninsula has three main floristic zones, which are warm-temperate, temperate, and cold-temperate. The warm-temperate zone prevails on the southern coast and islands, and is occupied by broadleaved evergreen species. The temperate zone covers the great majority of the Peninsula, away from the southern coast and high mountains, and is dominated by the Korean pine and various deciduous trees. Cold-temperate vegetation, including alpine and subalpine coniferous and broadleaved evergreen species, is found in North Korea and in the high mountains.

### Species data

**Fig 2:**
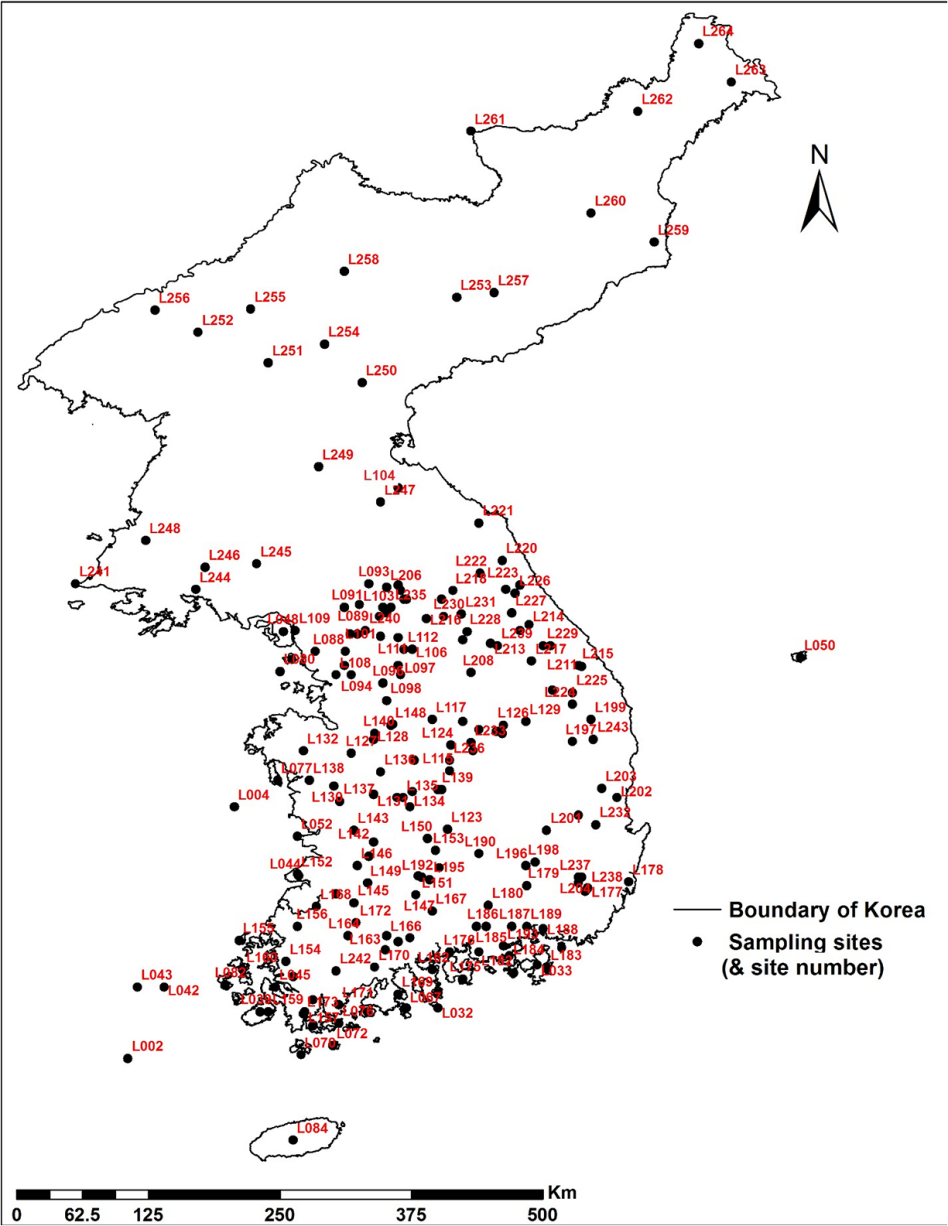
Sampling sites of cold-evergreens in the Korean Peninsula. Presence/absence on the sampling sites were obtained from Koo (2000). Sampling sites close to each other were separated into test data set and training data set to avoid biases from autocorrelation among data.

Presence and absence of twelve cold-evergreen species along the Korean Peninsula (Table 1) were obtained from Koo (2000) [40] (Fig 2 and See S1 Fig and S1(A) & S1(B) Table). A total of 182 sites of South Korea were visited to record floristic composition over the past forty years. Data for North Korea (22 sites) were obtained from the literatures [39, 41, 42]. The data of North Korea include investigations from the early 1900s to 1965. Recent data for North Korea are not available due to political reasons. The historical data were judged to be highly reliable according to Koo *et al* (2001). The data were collected by qualified botanists with the purpose of generating complete species lists in each site [40]. Thus the dataset can be regarded as having reliable absence data, which is sometimes a challenge for SDM modeling. Twelve cold-evergreens are found in North Korea and high mountains in the Korean Peninsula. All cold-evergreens are rare species and have been found in less than five sampling sites in the Korean Peninsula (S1(B) Table). In particular, *Diapensia lapponica* var. *Obovata*m, *Rhododendron aureum* and *Ledum palustre* var. *diversipilosum* were found at only one sampling site, Jeju Island, the southernmost part of the Korean Peninsula. Due to insufficient presence data for individual species (Table 1), we developed a single SDM for all cold-evergreens at a physiognomic-level in the Korean Peninsula.

**Table 1:** Species information of the cold evergreen broadleaved woody plants in the Korean Peninsula.

Species	# of sites with each species
*Ledum palustre* var. *maximum* Nakai	1
*Ledum palustre* var. *decumbens* Aiton	1
*Ledum palustre* var. *angustum* N. Busch	1
*Rhododendron lapponicum* subsp. *parvifolium* var. *parvifolium* (Adams) T.Yamaz	2
*Phyllodoce caerulea* (L.) Bab.	2
*Linnaea borealis* L.	2
*Ledum palustre* var. *diversipilosum* Nakai	6
*Rhododendron aureum* Georgi	9
*Rhododendron brachycarpum* D.Don ex G.Don	18
*Vaccinium vitis-idaea* L.	12
*Empetrum nigrum* var. *japonicum* K.Koch	1
*Diapensia lapponica* var. *obovata* F.Schmidt	1

### Climate data

We considered 19 bioclimatic variables and four seasonal climate variables, seasonal mean temperatures, assumed to be important for determining the distribution of plants [43] (Table 2). We obtained maps of 19 bioclimate variables and seasonal climate variables for current and future conditions from the WorldClim Dataset [44]. The current climate conditions were estimated by averaging for the period of 1950 to 2000. The future climate condition in 2050 were estimated by averaging for the period of 2040 to 2059, and the condition in 2070 for the period of 2069 to 2079 (http://www.worldclim.org/). The spatial resolution of climate data set is 1 km^2^.

**Table 2:** 23 climate variables: 19 bioclimate and 4 seasonal mean temperature variables considered in this study.

BIO1 = Annual Mean Temperature
BIO2 = Mean Diurnal Range (Mean of monthly (max temp—min temp))
BIO3 = Isothermality (BIO2/BIO7) (* 100)
BIO4 = Temperature Seasonality (standard deviation *100)
BIO5 = Max Temperature of Warmest Month
BIO6 = Min Temperature of Coldest Month
BIO7 = Temperature Annual Range (BIO5-BIO6)
BIO8 = Mean Temperature of Wettest Quarter
BIO9 = Mean Temperature of Driest Quarter
BIO10 = Mean Temperature of Warmest Quarter
BIO11 = Mean Temperature of Coldest Quarter
BIO12 = Annual Precipitation
BIO13 = Precipitation of Wettest Month
BIO14 = Precipitation of Driest Month
BIO15 = Precipitation Seasonality (Coefficient of Variation)
BIO16 = Precipitation of Wettest Quarter
BIO17 = Precipitation of Driest Quarter
BIO18 = Precipitation of Warmest Quarter
BIO19 = Precipitation of Coldest Quarter
Spring Mean Temperature
Summer Mean Temperature
Fall Mean Temperature
Winter Mean Temperature

Spring mean temperatures were calculated by averaging daily temperatures from March to May, summer mean temperature from June to August, fall mean temperature from September to November, and winter mean temperature from December to February.

### Climate change model selection

We used the future climate conditions of the WorldClim datasets projected with the Earth System configuration of the Hadley Centre Global Environment Model version 2 (HadGEM2-ES) under the Representative Concentration Pathways (RCP) 4.5 and RCP 8.5 scenarios. The observed increment of mean annual temperature in Korea over the last fifty years (0.23°C per decade) [45] is nearly twice that for the global mean annual temperature (0.13°C per decade) [46]. According to this warming trend in Korea, the RCP 4.5 projection is more realistic than the RCP 2.6 projection in explaining minimum effect of climate change on the distribution of cold evergreens.

HadGEM2 is the product of modeling the physical climate and adding earth system components and couplings. Two key features of physical performance targeted by the HadGEM2 family are El Niño Southern Oscillation (ENSO) and northern continent land-surface temperature biases. Therefore, the physical climate in the HadGEM2 family can sustain a realistic vegetation distribution, especially trees distributions [47]. In particular, the HadGEM2-ES model features the terrestrial and oceanic ecosystems and tropospheric chemistry, which simulate the carbon cycle in interactions with climate and improve predictions in vegetation distribution [48]. RCP 4.5 was developed by the GCAM (Global Change Assessment Model) modeling team at the Pacific Northwest National Laboratory's Joint Global Change Research Institute (JGCRI) (http://www.globalchange.umd.edu/models/gcam/) and RCP 8.5 by the MESSAGE modeling team and the IIASA Integrated Assessment Framework at the International Institute for Applies Systems Analysis (IIASA) [49]. RCP 4.5 assumed that the total radiative forcing was stabilized before 2100 by employing technologies and strategies which reduced greenhouse gas emissions [50]. RCP 8.5 projected the future climates under high greenhouse gas concentrations [50].

### Model Development

Data from the 204 sites were divided into 102 training points for model calibration and 102 test points for model evaluation. We used a multimodel inference, MMI, approach for developing SDM of cold-evergreens [51]. We used Pearson’s r correlation on pairs of predictors to eliminate the weaker predictors which showed high correlations (> 0.70) with another variable from the global model.

We built a set of candidate models with generalized linear model (GLM) assuming a binomial probability distribution (logistic regression) with the selected variables to predict site occupancy from all possible combinations of parameters contained in the global model. We calculated Akaike’s Information Criteria (AIC; Akaike 1973) with the small-sample bias adjustment (AICc) [38] to evaluate the fit of each candidate model for the MMI approach. Then we assessed the relative fit of each candidate model by calculating Akaike weights [38]. Following Burnham and Anderson (2002), model-selection uncertainty was incorporated by computing model-averaged estimates of the model coefficients with standard errors. We weighted parameter estimates and corresponding standard errors from each candidate model by that model’s associated Akaike weight and summed across the different models to develop a composite model. Probability of occurrence was calculated with the composite model and then back-transformed to the scale of the response variable.

Uncertainty in the classic model-average predictions was quantified by estimating confidence intervals (CI) and standard errors based on asymptotic normality [38]. However, the normal approximation is not appropriate for predictions of binary variables [38]. Therefore, following Hartley et al. (2006), we quantified uncertainty in model-averaged predictions with a total variance (*S* ^*2*^_*T*_), which is estimated by adding the weighted average of within-model variation to the between-model variation. Confidence intervals of the model-average predictions were estimated with 95% CI = Model-average predictions ± 1.96 *S*_*T*_. Uncertainty of predictions were identified by mapping the absolute difference between the upper and lower CI limits of estimates and the ratio of between-model to within-model variation on the response variable scale. The MMI analysis was implemented with the AICcmodavg package for R.3.0.0 [52], and spatial modeling with ArcGIS 10.1 (ESRI Inc., Redlands, CA).

### Model evaluation

Model performance was evaluated with the area under the curve (AUC) values of receiver operating characteristic (ROC) curves [53], the kappa statistic [54] and true skill statistic (TSS). The AUC value, the kappa statistic and TSS were calculated using the test data points. An ideal measure of SDM performance should not be influenced by the size of the specific data set (prevalence) but integrate sensitivity and specificity [55]. The sensitivity (omission error) is the proportion of observed presences correctly predicted by a model, and the specificity (commission error) the proportion of observed absences correctly predicted by a model [53]. However, the kappa statistic has been criticized due to its dependency on prevalence [56]. AUC is not dependent on prevalence but criticized because it equally weights omission and commission errors, ignores the actual probability values, which are important but not sensitive to transformation of the predicted probability, and depends on a geographic extent [57]. In particular, as expanding the geographical extent outside the present range, AUC values increase [57]. Therefore, performance of SDMs for rare species, such as cold-evergreens, found in a spatially restricted area can be overestimated by AUC values and underestimated by the kappa. Thus, we used TSS as well as kappa and AUC to improve model validation. TSS (sensitivity + specificity– 1) has been used as an alternative criterion for validating SDM performance because it accounts for commission and omission errors but is not affected by prevalence [55]. The current geographical range of cold-evergreens was projected by selecting a threshold of occurrence where sensitivity was equal to specificity [53, 58]. The ROC curve and kappa statistical analyses were implemented with the PresenceAbsence package for R.3.0.0 [59].

## Results

### Projections of cold-evergreens’ distribution in the Korean Peninsula

The Pearson's correlation analyses among 23 bioclimatic variables (S2D Table) identified six variables, BIO1, BIO2, BIO3, BIO12, BIO13 and BIO14, with weak correlations among them but high correlations with other climate variables (*r* < 0.7) (S2 Table). In particular, annual mean temperature (BIO1) had very strong correlations with most temperature variables except BIO2 and BIO3 where Pearson’s correlation coefficients varied from 0.72 to 1.00. We built a set of candidate models with all possible combinations of six variables and specified seven models consisting of 95% confidence set for the best model (Table 3). The 95% confidence set was determined by summing the Akaike weights of the ranked models until it was reached 0.95 for a 95 percent set [60]. Multimodel-average estimates (MMA) showed negative correlations with annual mean temperature, isothermality and precipitation of wettest month and positive correlations with mean diurnal range and precipitation of driest month (Table 4). Annual mean temperature was a dominant driving factor in model-average estimates (Table 4).

**Table 3:** Summary of MMI model selection statistics for the set of candidate models (*i*) predicting presence of cold-evergreens and (b) the model averaged estimate for each parameter.

Model	K	AICc	Δ AICc	*wi*
BIO1 + BIO14	3	48.04	0	0.25
BIO1 + BIO2	3	48.05	0.01	0.25
BIO1 + BIO13 + BIO14	4	48.8	0.77	0.17
BIO1 + BIO12 + BIO2	4	49.57	1.53	0.12
BIO1 + BIO2 + BIO3	4	49.8	1.76	0.1
BIO1 + BIO12 + BIO13 + BIO14	5	50.87	2.83	0.06
BIO1 + BIO12 + BIO2 + BIO3	5	51.74	3.7	0.04

Symbols: AIC = Akaike information criteria, AICc = The second order information criterion, small sample unbiased AIC, (AICc) [79], Δ AICc = Difference from the smallest AICc, *w*_*i*_ = Akaike weights of the candidate model *i*.

**Table 4:** Summary of MMI model selection statistics for the model averaged parameter estimates.

Parameter	Model-average estimate	Unconditional SE	95% unconditional CI Lower limit	95% unconditional CI Upper limit
BIO1	-0.08	0.02	-0.12	-0.04
BIO2	-0.13	0.05	-0.23	-0.04
BIO3	0.14	0.28	-0.41	0.69
BIO12	0	0.05	-0.09	0.09
BIO13	-0.01	0.01	-0.03	0.01
BIO14	0.13	0.06	0.01	0.25

Symbols: SE = Standard error, and CI = Confidence interval.

The performance of MMA was excellent based on the AUC value, 0.95 (Tables 5 & 6). The AUC value of 0.95 indicates the excellent agreement between the predicted and the observed ranges [55, 61]. The kappa statistics was 0.62 (*P* < 0.001) [54], and TSS 0.77 [55] (Table 6). Both values also indicated a good performance of MMA.

**Table 5:** A confusion matrix.

		Observed	
		Presence	Absence
Predicted	Presence	12	8
	Absence	2	80

**Table 6:** Model validation statistics.

Omission errors (Sensitivity)	0.86
Commission errors (Specificity)	0.91
TSS	0.77
AUC	0.95
Kappa	0.62

Symbols: TSS = True skill statistics, AUC = Area under the curve.

**Fig 3:**
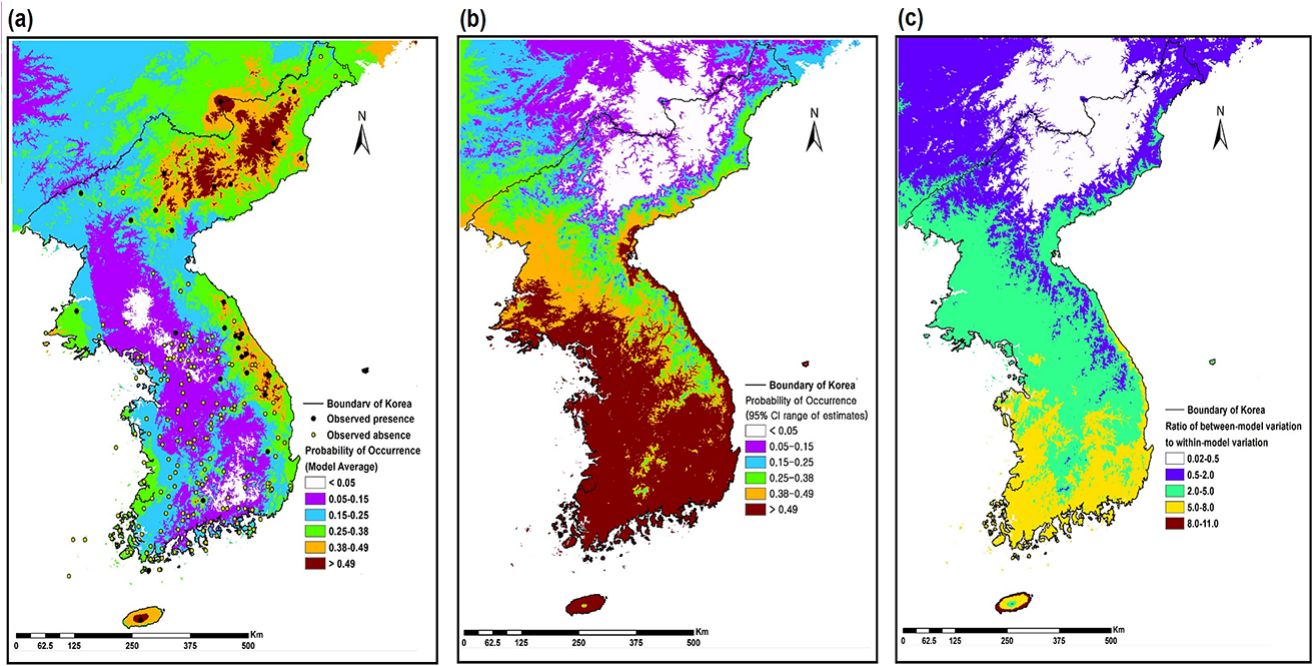
(A) Mean climatic suitability of the cold-evergreens according to the multimodel weighted average; (B) 95% confidence interval (CI) of estimates; (C) ratio of between-model variation to within-model variation.

Climatic suitability projected by the MMA model increased from the southwest, lowland areas, to the northeast, high mountains, showing the highest suitability in the northeastern Peninsula and the lowest in the southeastern Peninsula (Fig 3(A)). The 95% CI for projected suitability explained that greatest uncertainty existed over predictions for the southwest and lowland areas and the lowest uncertainty in the northeastern Peninsula and high mountain areas in the southeast (Fig 3(B)). In general, the variation between models was two or more times that of the within-model variation except in the areas of the northeastern Peninsula and near the mountain-tops in northeast, southeast and Jeju island (Fig 3(C)). The geographic range of cold-evergreens was projected based on a threshold, 0.38, where sensitivity was equal to specificity. The orange and red colored areas show the relative suitability for cold-evergreens in Fig 3(A).

### Geographical distribution of cold-evergreens under climate change

**Fig 4:**
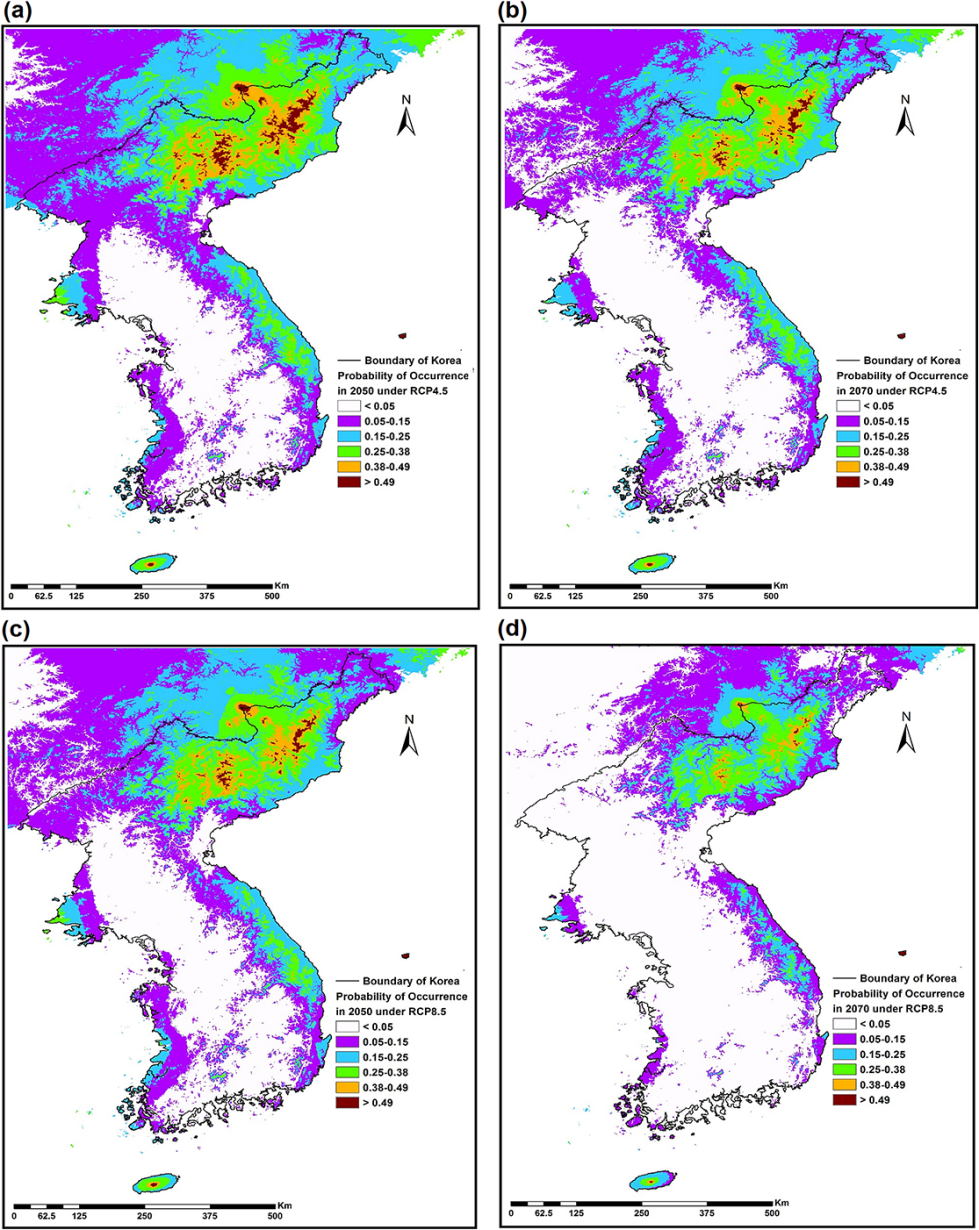
Climate Change effects on the cold-evergreens' distributions in the Korean Peninsula. The suitability projection of cold-evergreens according to the multimodel weighted average in 2050 under RCP4.5 scenario (a); in 2070 under RCP 4.5 (b); in 2050 under RCP 8.5 (c); and in 2070 under RCP 8.5 (d).

**Fig 5:**
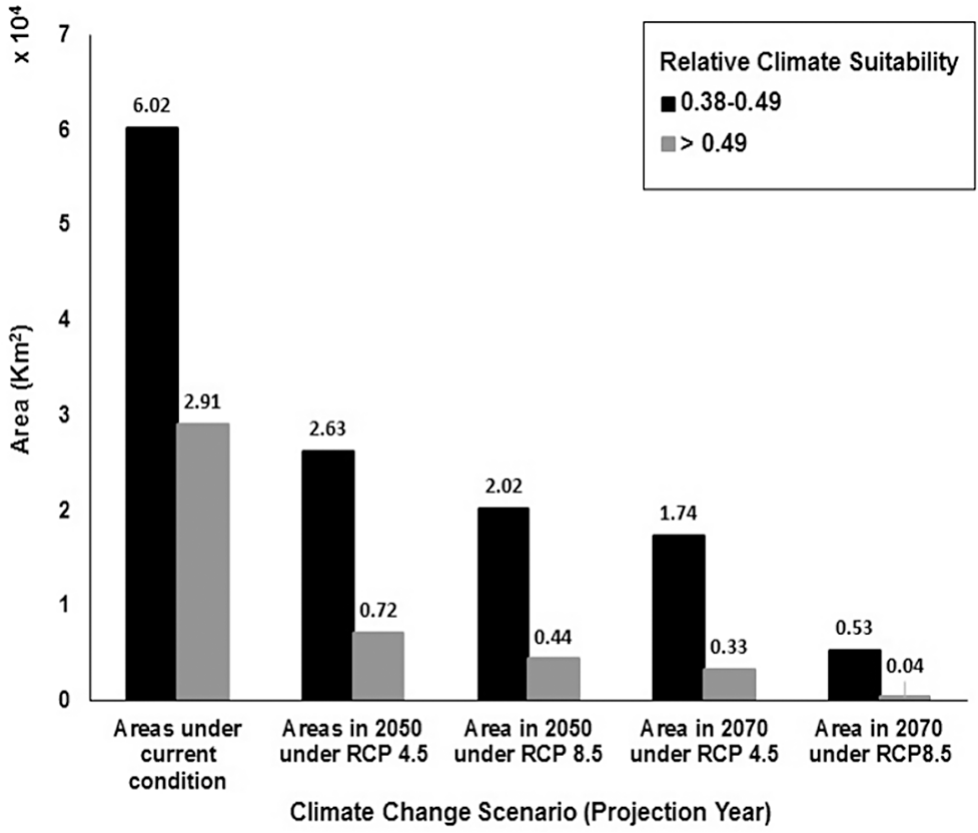
Climate change effects on the areal changes of cold-evergreens. The geographical ranges were predicted using the sensitivity = specificity threshold of 0.38 under the current and future climate conditions.

Climate change effects on the geographical range of cold-evergreens in 2050 and 2070 were predicted under RCP 4.5 and RCP 8.5 scenarios (Fig 4A–4D). 89,265 km^2^ were predicted to be climatically suitable using the sensitivity = specificity threshold of 0.38 under the current climate condition. Climatically suitable areas were reduced to 33,498 km^2^ in 2050 and 20,726 km^2^ in 2070 under the RCP 4.5 projection and 24,624 km^2^ in 2050 and 5,717 km^2^ in 2070 under the RCP 8.5 projection (Fig 5, S3 Table). Cold-evergreens could lose 62.47% of their climatically suitable habitat in 2050 and 76.78% in 2070 under the RCP.4.5 projection, 72.41% in 2050 and 93.60% under the RCP.8.5 projection (S3 Table). The geographical range in 2050 and 2070 under the RCP 4.5 projection and in 2050 under RCP 8.5 projection shrunk to near mountain-tops in the south (ROK), but most of the area in the north (DPRK) remained suitable (Fig 4(A), 4(B) & 4(C)). All cold-evergreens disappeared in the south except near the mountain-top on Jeju island, the southernmost part of the south (ROK), and high-elevation habitats in North Korea under the RCP 8.5 projection (Fig 4(D)).

## Discussion

With rapid and directional global environmental changes during the past decade, ecologists have increased their focus on the potential responses of plant communities to climate change [62]. Range shrinkage and extinction of organisms have been reported in relation to climate change [2, 4–6, 63]. We have examined how climatic factors impact climatic habitat suitability and geographic range of cold-evergreens in the Korean Peninsula.

Our results showed that annual mean temperature was a dominant driving factor and showed a negative correlation with the range of cold-evergreens (Table 4). Precipitation was a minor factor, and precipitation of driest month showed a positive correlation with the range of cold-evergreens. The negative correlations with temperature indicate photosynthetic and respirational costs under warmer temperature for the cold-evergreens. Previous research explained possible ecological processes for the negative effects of warmer temperature on alpine and subalpine species. These ecological processes included 1) reduced growth period by increasing winter dormancy period [3, 64], 2) increased late-season frost damage to buds released from dormancy under warmer temperature, 3) wintertime drought damage in combination with frozen soil [65], and 4) increased high temperature-induced photoinhibition during summer [19, 66]. Sufficient annual precipitation in the Korean Peninsula [39, 67] may account for the minor effect of precipitation. However, precipitation of driest month could enhance the warmer temperature effect because drought stress intensifies the negative effects of warmer temperature on photosynthesis [68] (Table 4). These relationships are well supported by previous research showing that these species, alpine and subalpine dwarf shrubs, are sensitive to warmer temperatures [31, 66, 69]. It has been observed that alpine and subalpine dwarf shrub communities have declined under climatic warming [31–33]. The 50-year survey showed that montane dwarf shrubs and heaths significantly lost their suitable habitats under climate warming [31, 70].

Our model predicted high vulnerability of the cold-evergreens to potential future climatic warming: 62.47% habitat loss in 2050 76.78% in 2070 under the RCP 4.5 projection and 72.41% in 2050 and 93.60% under the RCP 8.5 projection. In particular, the cold-evergreens were projected to disappear in the south ROK except Jeju island in 2070 under the climate change projection of RCP 8.5. The northward range shift of cold-evergreens under climate warming would cause zonal changes in species composition in the Peninsula under climate change. This shift may change the alpine and subalpine ecosystem structure and function in the Peninsula [71].

Our model predictions showed an excellent performance based on AUC (0.95), Kappa (0.62) and TSS (0.77) (Table 6). However, we are well aware that factors other than temperature and precipitation define niche space and that our simplistic models may not accurately predict the impact of climate change on the distribution of plants in the Korean Peninsula. Factors other than climatic tolerance that may also be important in defining future plant distributions include dispersal limitation [16, 72], interactions with coexisting species, and the ability of plants to adapt and acclimate [62]. Theoretical and field studies have supported dispersal limited distribution patterns of plants [73, 74]. Results of many transplant experiments have shown genetic differentiation along with environmental gradients and concluded that modern populations of species that shifted ranges in the past were adapted to the climatic conditions of their present habitats [62, 75, 76]. Hamrick (2004) suggested that trees might contain adequate genetic diversity through high gene-flow among populations to respond to changed climatic conditions.

Little ecological research has been implemented for the alpine and subalpine cold-evergreens in the Korean Peninsula. We first need to improve our understanding of the cold-evergreen ecosystems at a community level and an individual species level. It will increase accuracy of model predictability, which will offer better information for conservation policies and management of cold-evergreens. For this, much ecological research related to dispersal mechanisms, adaptation and acclimation potential, and interactions with coexisting species is needed for each cold-evergreen species at multiple spatial scales.

The predictions are also affected by model uncertainty [34, 51] and limitations of the measurement system, such as insufficient sample size, observation errors, and strategies of sampling and data collection [77, 78]. In particular, we have insufficient present-day presence/absence data from North Korea due to political reasons. It could increase model uncertainty. However, despite sufficient data from South Korea, uncertainty of predictions showed the greatest model uncertainty over predictions for the southwest and lowland areas (Fig 3(B)). Observation errors, strategies of sampling, and other ecological factors not considered in the model, may account for high uncertainty in southwest and lowland areas. Implementing ecological research for each cold-evergreen species at a local scale and development of new sampling strategies and techniques will decrease model uncertainty.

Alpine and subalpine rare, relict species have low genetic diversity and have shown lower acclimation and adaptation potential to climate warming than lowland trees [19, 25]. Accordingly, adaptation and acclimation related uncertainties could have less impact on our prediction of climate change effects on the range of cold-evergreens. Also, model uncertainty is lowest in the current range, the northeastern Peninsula and high mountain areas in the southeast (Fig 3(B)). Therefore, despite our shortage of ecological knowledge of cold-evergreens and high model uncertainty in southwest and lowland areas, our predictions can offer critical information about the potential climate change effects on cold-evergreens’ distribution in support of long-term conservation policies and management of cold-evergreens in the Korean Peninsula. In particular, suitable habitats, which were predicted by our model but not currently occupied by the cold-evergreens, could be refuges for the cold-evergreens under climate change. We may reduce overall habitat loss and population decline of cold-evergreens by transplanting them to refuges.

## Conclusion

Species distribution models (SDMs) are being widely used to predict spatiotemporal variations in plant and animal distributions. SDMs we developed for the Korean Peninsula identified climate factors describing the current geographical range of cold-evergreens. Annual mean temperature was a dominant factor and showed a negative correlation with the range. However, annual precipitation was a minor factor in the model-average estimates of cold-evergreen occupancy. SDMs showed excellent performances (AUC = 0.95, kappa = 0.62, TSS = 0.77). The cold-evergreens would lose 62.47% of their habitat in 2050 76.78% in 2070 under the RCP 4.5 projection and 72.41% in 2050 and 93.60% under the RCP 8.5 projection. Mountain-tops in the south and most of the area in the north remain suitable in 2050 and 2070 under the RCP 4.5 projection and 2050 under RCP 8.5 projection. Only high-elevations in the northeastern Peninsula remain suitable under the RCP 8.5 projection. This northward shift in range indicates changes in species composition at the alpine and subalpine ecosystems.

## Supporting Information

S1 Fig Sampling sites of cold-evergreens in the Korean Peninsula. (DOCX)

S1 Table (A) shows the list of cold-evergreens. (B) presence/absence on the sampling sites obtained from Koo (2000). Sampling sites close to each other were separated into test data set and training data set to avoid biases from autocorrelation among data.
(DOCX)

S2 Table Pearson’s correlations on a paired climate variables. (DOCX)

S3 Table Habitat shrinks under climate change: (A) Areal changes of each probability of occurrence under difference climate change scenarios; (B) Percent areal changes under difference climate change scenarios. (DOCX)
