# Supplementary figures and images for: Potential Effects of Climate Change on the Distribution of Cold-Tolerant Evergreen Broadleaved Woody Plants in the Korean Peninsula
Source: PLoS One. 2015 Aug 11;10(8):e0134043. doi: 10.1371/journal.pone.0134043 (PMC4532508; doi:10.1371/journal.pone.0134043)

**S1 Fig. Sampling sites of cold-evergreens in the Korean Peninsula.**


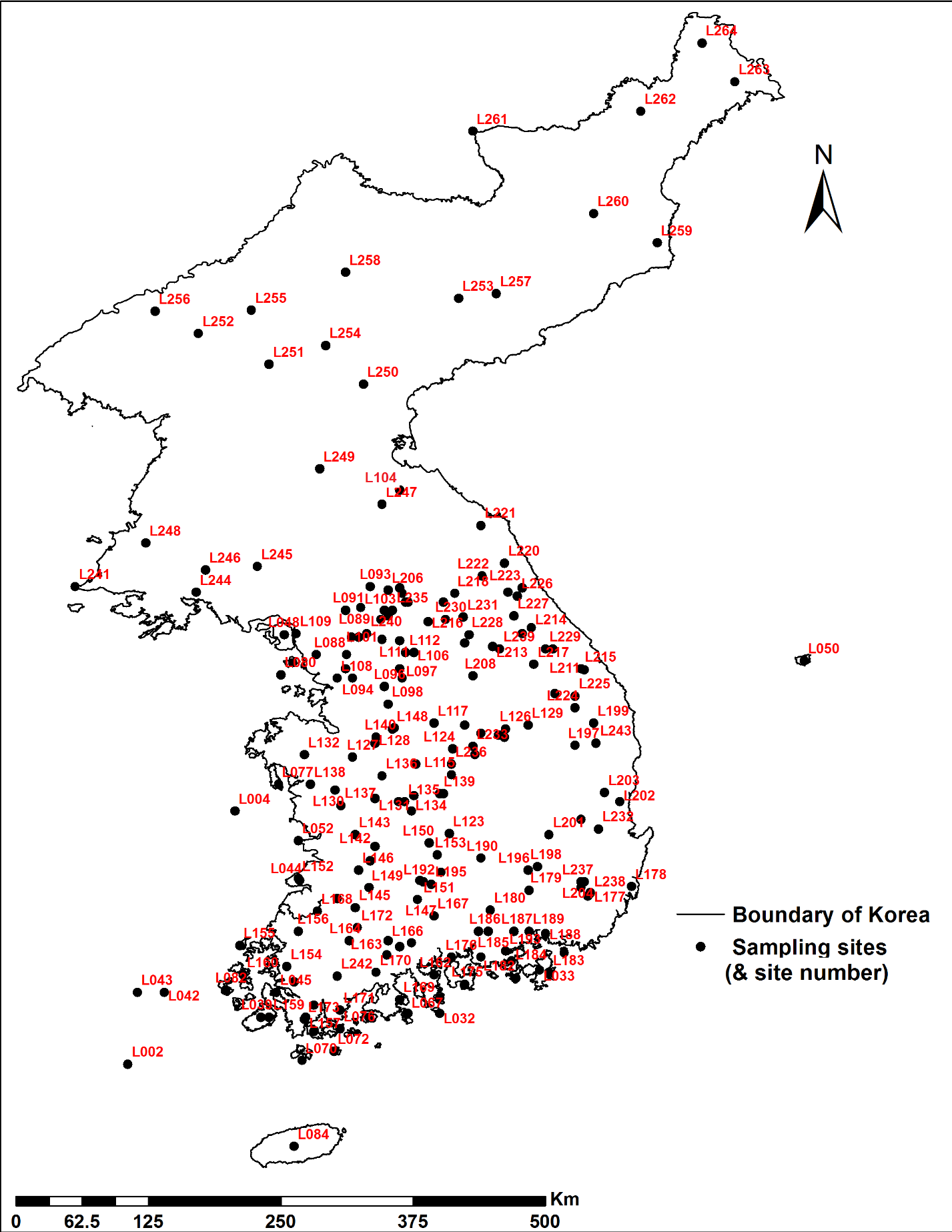

Supplement: S1 Fig — (DOCX) [file pone.0134043.s001.docx]
