# Supplementary material for: Potential Effects of Climate Change on the Distribution of Cold-Tolerant Evergreen Broadleaved Woody Plants in the Korean Peninsula
Source: PLoS One. 2015 Aug 11;10(8):e0134043. doi: 10.1371/journal.pone.0134043 (PMC4532508; doi:10.1371/journal.pone.0134043)
Supplement: S2 Table — (DOCX) [file pone.0134043.s003.docx]

**S2 Table. Pearson’s correlations on a paired climate variables.**
