# Supplementary material for: Potential Effects of Climate Change on the Distribution of Cold-Tolerant Evergreen Broadleaved Woody Plants in the Korean Peninsula
Source: PLoS One. 2015 Aug 11;10(8):e0134043. doi: 10.1371/journal.pone.0134043 (PMC4532508; doi:10.1371/journal.pone.0134043)
Supplement: S3 Table — (DOCX) [file pone.0134043.s004.docx]

**S3 Tables. Habitat shrinks under climate change: (a)** Areal changes of each probability of occurrence under difference climate change scenarios; **(b)** Percent areal changes under difference climate change scenarios.

**(a)**

| **Probability of Occurrence** | **Areas under current condition** | **Areas in 2050 under RCP 4.5** | **Area in 2050 under RCP 8.5** | **Area in 2070 under RCP 4.5** | **Area in 2070 under RCP8.5** |
| --- | --- | --- | --- | --- | --- |
| **< 0.05** | 11615 | 121495 | 157088 | 175747 | 307420 |
| **0.05-0.15** | 101213 | 159853 | 165260 | 153882 | 110048 |
| **0.15-0.25** | 176926 | 123489 | 109400 | 104737 | 59257 |
| **0.25-0.38** | 135994 | 77064 | 59038 | 60318 | 32968 |
| **0.38-0.49** | ***60166*** | ***26279*** | ***20241*** | ***17403*** | ***5307*** |
| **> 0.49** | ***29099*** | ***7219*** | ***4383*** | ***3323*** | ***410*** |
| **> 0.38** | ***89265*** | ***33498*** | ***24624*** | ***20726*** | ***5717*** |

**(b)**

| **% areal change under climate changes** | | |  |  |
| --- | --- | --- | --- | --- |
| **Probability of Occurrence** | **Areal change in 2050 under RCP4.5** | **Areal change in 2050 under RCP8.5** | **Areal change in 2070 under RCP4.5** | **Areal change in 2070 under RCP8.5** |
| **< 0.05** | 946.02 | 1252.46 | 1413.10 | 2546.75 |
| **0.05-0.15** | 57.94 | 63.28 | 52.04 | 8.73 |
| **0.15-0.25** | -30.20 | -38.17 | -40.80 | -66.51 |
| **0.25-0.38** | -43.33 | -56.59 | -55.65 | -75.76 |
| ***0.38-0.49*** | ***-56.32*** | ***-66.36*** | ***-71.08*** | ***-91.18*** |
| ***> 0.49*** | ***-75.19*** | ***-84.94*** | ***-88.58*** | ***-98.59*** |
| **> 0.38** | ***-62.47*** | ***-72.41*** | ***-76.78*** | ***-93.60*** |
